# Prehospital Care Under Fire: Strategies for Evacuating Victims from the Mega Terrorist Attack in Israel on October 7, 2023

**DOI:** 10.1017/S1049023X24000438

**Published:** 2024-06

**Authors:** Eli Jaffe, Ziv Dadon, Evan Avraham Alpert

**Affiliations:** 1. Community Division, Magen David Adom, Or Yehuda, Israel; 2. Department of Emergency Medicine, Faculty of Health Sciences, Ben-Gurion University of the Negev, Beer Sheva, Israel; 3. Jesselson Integrated Heart Center, Shaare Zedek Medical Center, Jerusalem, Israel; 4. Faculty of Medicine, Hebrew University of Jerusalem, Israel; 5. Department of Emergency Medicine, Hadassah University Hospital, Jerusalem, Israel

**Keywords:** disasters, Emergency Medical Services, Israel, mass-casualty incidents, terrorism

## Abstract

On October 7, 2023, somewhere around 1,500-3,000 terrorists invaded southern Israel killing 1,200 people, injuring 1,455, and taking 239 as hostages resulting in the largest mass-casualty event (MCE) in the country’s history. Most of the victims were civilians who suffered from complex injuries including high-velocity gunshot wounds, blast injuries from rocket-propelled grenades, and burns. Many would later require complex surgeries by all disciplines including general surgeons, vascular surgeons, orthopedists, neurosurgeons, cardiothoracic surgeons, otolaryngologists, oral maxillofacial surgeons, and plastic surgeons. Magen David Adom (MDA) is Israel’s National Emergency Prehospital Medical Organization and a member of the International Red Cross. While there are also private and non-profit ambulance services in Israel, the Ministry of Health has mandated MDA with the charge of managing an MCE. For this event, MDA incorporated a five-part strategy in this mega MCE: (1) extricating victims from areas under fire by bulletproof ambulances, (2) establishing casualty treatment stations in safe areas, (3) ambulance transport from the casualty treatment stations to hospitals, (4) ambulance transport of casualties from safe areas to hospitals, and (5) helicopter transport of victims to hospitals. This is the first time that MDA has responded to a mega MCE of this magnitude and lessons are continually being learned.

## Specific Event Identifiers

- **Event Type** Mega Mass-Casualty Terrorist Attack
- **Event Onset Date** October 7, 2023
- **Location of Event** Southern Israel bordering Gaza, including between Zikim, Sderot, Netivot, and Kerem Shalom
- **Geographic Coordinates** 31°36'35.4"N 34°31'15.4"E 28m; 31°31'22.1"N 34°35'44.1"E 95 m; 31°25'24.2"N 34°35'42.1"E 154 m; 31°13'44.3"N 34°17'03.6"E 88m
- **Dates of Observation Reported** October 7-8, 2023
- **Response Type** Prehospital Emergency Medical Service

## Introduction

Any mass-casualty event (MCE) poses significant challenges to the overall health care system, and in particular, to a prehospital emergency medicine organization.^1,2^ Unfortunately, Israel has had to deal with terrorist attacks on its civilian population as well as numerous cross-border conflicts with terrorist organizations in Gaza over the last two decades.

The largest MCE in the country’s history took place on October 7, 2023 when between 1,500-3,000 terrorists from Hamas, Palestinian Islamic Jihad, and other organizations invaded southern Israel killing 1,200 people, injuring 1,455, and taking 239 as hostages.^3–5^ The incident began at 06:30 with a massive missile attack as a diversionary operation. A few minutes later, a ground attack began by terrorists equipped with automatic weapons and rocket-propelled grenades resulting in many emergent casualties within the initial hours of the event. These included individuals suffering from high-velocity bullet wounds, blast injuries, and burns. The event spread over 30 towns, villages, and kibbutzim (communal villages) across hundreds of square kilometers, as well as three cities with 100,000 residents. Much of the area, including main roads, was besieged and it was often not possible to reach or evacuate casualties for hours. The objective of this field report is to describe the response of Israel’s National Emergency Prehospital Medical Organization to this mega MCE and the lessons learned.

### Magen David Adom

Israel’s Ministry of Health (Jerusalem, Israel) has mandated Magen David Adom (MDA; Or Yehuda, Israel) – Israel’s National Emergency Prehospital Medical Organization and a member of the International Red Cross (Geneva, Switzerland) – to manage all MCEs. While there are also private and non-profit ambulance services in Israel, they operate under the supervision of MDA in an MCE. The organization routinely operates 200 ambulance stations nation-wide and up to 500 medical teams around the clock. Treatment is based on the Anglo-American approach with emergency vehicles divided into two levels – Advanced Life Support (ALS) teams managed by paramedics and Basic Life Support (BLS) teams managed by emergency medical technicians (EMTs). Currently, MDA has approximately 3,500 employees, 30,000 volunteers, and operates 1,450 vehicles. This includes 20 bulletproof ambulances (additional metal plating to protect against bullets and shrapnel), emergency motorcycles, three helicopters (as part of Israel’s helicopter Emergency Medical Services [HEMS], which on a civilian level is also serviced by the military), and 24 mass-casualty response vehicles. These large vehicles serve as a storage unit for additional medical supplies and equipment both at the level of BLS and ALS. They can also be transformed into an ad-hoc treatment site.

In an MCE, triage is performed at the scene of the incident and then the victims are quickly evacuated to hospitals – based on the concept of “save and run” – to provide only immediate life-saving treatment such as the application of a tourniquet for limb bleeding or needle application for a tension pneumothorax. All teams working at MDA are trained and updated in MCE response.

## Observations

When the attack began, approximately 400 teams were on duty throughout the country, of which around 15 teams were at five stations near the border with Gaza and an additional 15 ambulances were on call in nearby towns. Immediately after the start of the missile attack, MDA raised the level of alert and staffing of the ambulances. There were 300 personnel on the overnight shift, and by 07:00, MDA increased the number of staff to 960. This was the time that the shifts changed so that those overnight stayed on, staff from the new shift started, and those hearing of the events rushed to the stations. By the following hour, all ambulances and rescue vehicles were manned.

Approximately 150 ambulances and rescue vehicles headed from the center of the country towards the conflict area with non-stop rocket barrages overhead. The situation became complicated as in the early hours of the terrorist attack, three MDA staff were killed – one paramedic, an ambulance driver, and an on-call volunteer first responder. In addition, early on, four ambulances were intentionally shot and damaged, putting them out of commission.^6^

For this mega MCE, MDA incorporated a five-part strategy: (1) extricating victims from areas under fire by bulletproof ambulances, (2) establishing casualty treatment stations in safe areas, (3) ambulance transport from the casualty treatment stations to hospitals, (4) ambulance transport of casualties from safe areas to hospitals, and (5) helicopter transport of victims to hospitals.

Out of 20 bulletproof ambulances operated by MDA, only one is stationed near the Gaza area. Shortly after 07:00 in the morning, one-half of the protected ambulances headed towards the south of the country. These reinforced ambulances entered the battle zones, rapidly gathered victims, shuttled them to the casualty treatment stations, and quickly returned for more victims. Treatment in the battle zones consisted mostly of stopping the bleeding with bandages and tourniquets, and bag-valve-mask ventilation, if necessary.

**Figure 1.**
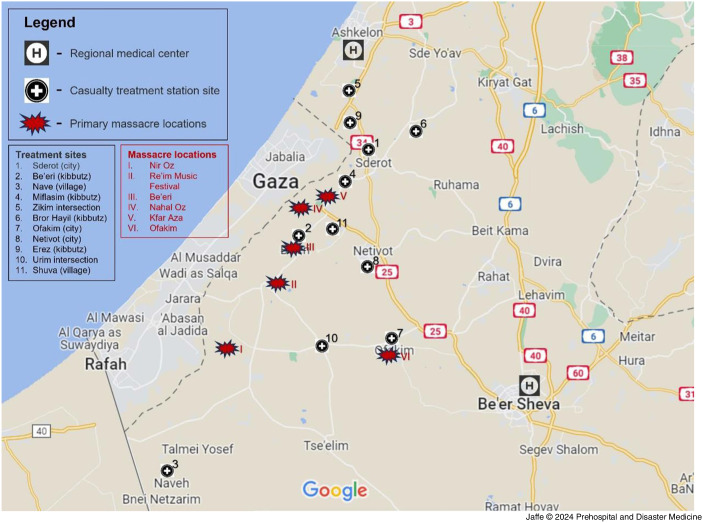
Map of Casualty Treatment Stations in Relation to Main Massacre Sites. Note: From Google Maps (Google Inc.; Mountain View, California USA).

The goal of the casualty stations was to triage and stabilize the victims prioritizing the most emergent. The stations were mostly staffed by EMTs and paramedics, although some had physicians. Procedures included intubations, needle application for tension pneumothorax, intravenous line insertion, and tourniquet placement. The staff also administered intravenous fluids, tranexamic acid, pain medications including ketamine, and plasma. Many of the victims would later require complex surgeries by all disciplines, including general surgeons, vascular surgeons, orthopedists, neurosurgeons, cardiothoracic surgeons, otolaryngologists, oral maxillofacial surgeons, and plastic surgeons. Three of the sites were MDA stations and one was a dental clinic. Other indoor locations included a synagogue, a kibbutz safe room, and a private house. Four other casualty stations were placed on the roadside, as these were at critical junctions between the areas of evacuation and the hospitals. Overall, MDA treated and stabilized 306 victims at the stations (Table 1 and Figure 1). In a limited number of cases, the bulletproof ambulances evacuated the victims directly to the hospitals. Approximately 100 rounds were performed by the bulletproof ambulances.

**Table 1.** Casualty Treatment Stations Established during the First Hours of the October 7, 2023 Terror Attack Including Locations, Operating Hours, Number of Victims Treated, Teams, and Available Equipment

Casualty Treatment Station Site	Location	Operating Hours	Number of Casualties	Team	Equipment
Sderot (city)	MDA Station	07:00-13:00	6	Two paramedics and two EMTs	ALS ambulance and mass-casualty emergency storage
Be’eri (kibbutz)	Dental Clinic	07:00-14:30	12	One physician and one paramedic	From clinic and from on-call volunteer first responder
Nave (village)	Synagogue	07:10-16:00^a^	12	Three paramedics and three EMTs	ALS ambulance and from helicopter
Miflasim (kibbutz)	Private House	07:40-12:00	4	One paramedic and one EMT	ALS ambulance
Zikim Intersection	Roadside	08:00-20:00	13	Teams from six ALS ambulances and seven BLS ambulances	ALS ambulance
Bror Hayil (kibbutz)	Roadside	08:30-01:00^a^	71	Teams from six ALS ambulances and 15 BLS ambulances	ALS ambulance and mass-casualty vehicle
Ofakim (city)	MDA Station	08:30-17:30	80	Paramedics and EMTs	ALS ambulance and mass-casualty emergency storage
Netivot (city)	MDA Station	08:30-17:00	11	One paramedic and one EMT	ALS ambulance and mass-casualty vehicle and emergency storage
Erez (kibbutz)	Safe Room	09:00-12:30	4	One paramedic	From clinic and from on-call volunteer first responder
Urim Intersection	Roadside	16:30-18:30	13	One physician, two paramedics, and EMTs	ALS ambulance
Shuva (village)	Roadside	18:30-06:00^a^	80	One physician, one paramedic, and EMTs	Three ALS ambulances and four BLS ambulances

Abbreviations: ALS, Advanced Life Support; BLS, Basic Life Support; EMT, emergency medical technician; MDA, Magen David Adom.

a The operating hours started on October 7, 2023 and ended on October 8, 2023.

Currently, MDA has three helicopters, which evacuated 21 casualties from the area to hospitals in the center of the country. In the afternoon of October 7, one helicopter was hit by gunfire and had to make an emergency landing. The crew was not injured but the helicopter was out of action.

Additional victims who arrived either on their own or by car to an area out of the battle zone met up with MDA and were transported by ambulances directly to hospitals. Private ambulance services, non-profit ambulance services, the army, or private vehicles also evacuated victims.

On October 7, 2023, MDA was involved in transporting over 700 victims directly to hospitals, as well as dozens of others to army helicopters which then flew to medical centers throughout the country.

## Analysis

The mega terrorist attack that occurred in Israel on October 7, 2023 was essentially a dynamic on-going combat zone but involved mostly civilian victims. While there is much literature on advances in treatment on the battlefield, such as tourniquet application^7^ or the early deployment of whole blood,^8^ there is less research on evacuation strategies of a mega MCE in the civilian environment. The literature often uses different terminology with the area under attack known as either the “inner cordon,” “non-permissive environment,” “hot zone,” or “danger zone.” The area outside of the line of fire is known as the “outer cordon,” “permissive environment,” “cold zone,” or “safe zone.” Some literature discusses the semi-permissive environment as the area in between the non-permissive and permissive environments and is established after the threat is suppressed. Treatment in this area is still limited.^9^ In previous descriptions of marauding or multisite terrorist attacks, the danger zones were relatively localized and limited. For example, in the November 13, 2015 terrorist attack in Paris, France, there were four sites where shootings took place and three where there were bombs. In the area of the Bataclan Music Hall, tactical physicians entered the danger zone where they applied tourniquets and wound dressings and moved victims to treatment areas in the safe zones.^10,11^ Another example of a dynamic terrorist attack was the mass shooting on Utoya Island, Norway in 2011. In that event, 69 people were killed by a lone gunman on an island that could only be reached by ferry. The initial ambulances were halted when bullets were noted in the water. Helicopters were also prevented from landing on the island. It was only later that a casualty treatment station could be set up on the island. Eventually, the wounded could be evacuated from the scene, treated by Emergency Medical Services (EMS), and transported to hospitals.^12^

During the mega MCE on October 7, 2023, the area under attack was spread over hundreds of square kilometers and was dynamic as the terrorists were continually on the move in trucks and on motorcycles. This sometimes led to the shifting of the delineation between the danger zones and the safe zones. There have been 184 terrorist attacks against EMS reported in the Global Terrorist Database,^13^ and on October 7, 2023, MDA was in the middle of a mega MCE and part of all soft targets in the area.

Similar to many MCEs, casualty treatment stations were set up in the safe zones. In combat situations, the United States military employs the use of the battalion aid station, which is the first stop past the combat medic. However, in recent military conflicts, these have been mostly bypassed due to the ability of air transport to more advanced facilities.^14^ On October 7, 2023, while most victims were transported by ambulance to hospitals, a limited number were flown by HEMS.

## Conclusion

While MDA has responded to hundreds of MCEs, this event was unique in several aspects: (1) the evacuations occurred for mostly civilian casualties during an on-going attack by well-armed terrorists, (2) the high number of victims (over 1,000), and (3) the complexity of their wounds including penetrating trauma from high-velocity gunshot wounds, blast injuries, and burns. Even some of the teams that were operating outside of the area with heavily armed terrorists were still in the line of rocket fire. Because of the constant danger surrounding the event and the need for rapid evacuation, not every casualty could be recorded. The exact number of transported victims is still unknown.

There are clear lessons learned from October 7, 2023 that can be incorporated into the response to a mega MCE that is on-going and dynamic. Flexibility is essential in terms of the ability to move forces to areas where the wounded are located as well as to areas of safety. The use of bulletproof ambulances in the danger zones to shuttle patients to the safe zones was critical in saving the lives of the victims as well as protecting the staff. In addition, casualty treatment stations proved vital as they allowed areas where the wounded could be stabilized and the non-bulletproof ambulances could then make rapid transports to hospitals. Afterward, MDA is still evaluating its actions from this unique event and developing and refining its protocols to prepare for future MCEs.
